# Supplementary material for: Multi‐dataset identification of innovative feature genes and molecular mechanisms in keratoconus
Source: J Cell Mol Med. 2024 Sep 19;28(18):e70079. doi: 10.1111/jcmm.70079 (PMC11412914; doi:10.1111/jcmm.70079)
Supplement: Supplementary file 2 — Material S2. [file JCMM-28-e70079-s002.zip › 7.GSEA analysis/1.ARL11/KEGG_ALLOGRAFT_REJECTION.html]

Details for gene set KEGG\_ALLOGRAFT\_REJECTION[GSEA]

|  || Dataset | ARL11.group.cls#h\_versus\_l |
| Phenotype | group.cls#h\_versus\_l |
| Upregulated in class | l |
| GeneSet | KEGG\_ALLOGRAFT\_REJECTION |
| Enrichment Score (ES) | -0.46166578 |
| Normalized Enrichment Score (NES) | -1.1073507 |
| Nominal p-value | 0.35318276 |
| FDR q-value | 1.0 |
| FWER p-Value | 0.996 |
Table: GSEA Results Summary

  

Fig 1: Enrichment plot: KEGG\_ALLOGRAFT\_REJECTION      
 Profile of the Running ES Score & Positions of GeneSet Members on the Rank Ordered List

  

| SYMBOL | TITLE | RANK IN GENE LIST | RANK METRIC SCORE | RUNNING ES | CORE ENRICHMENT || 1 | HLA-DRB5 | na | 2778 | 0.251 | -0.0265 | No |
| 2 | GZMB | na | 2928 | 0.244 | 0.0213 | No |
| 3 | HLA-DOA | na | 3812 | 0.207 | 0.0401 | No |
| 4 | HLA-DRB4 | na | 7218 | 0.107 | -0.0352 | No |
| 5 | HLA-DRA | na | 7656 | 0.099 | -0.0268 | No |
| 6 | HLA-DPA1 | na | 7767 | 0.097 | -0.0093 | No |
| 7 | HLA-DRB1 | na | 7965 | 0.093 | 0.0049 | No |
| 8 | CD86 | na | 8057 | 0.091 | 0.0218 | No |
| 9 | HLA-DQB1 | na | 8352 | 0.086 | 0.0316 | No |
| 10 | HLA-F | na | 10625 | 0.052 | -0.0228 | No |
| 11 | IL12A | na | 13401 | 0.024 | -0.0977 | No |
| 12 | HLA-G | na | 14146 | 0.018 | -0.1154 | No |
| 13 | HLA-DPB1 | na | 14439 | 0.016 | -0.1205 | No |
| 14 | HLA-E | na | 14680 | 0.014 | -0.1245 | No |
| 15 | HLA-C | na | 15719 | 0.006 | -0.1532 | No |
| 16 | FASLG | na | 15958 | 0.004 | -0.1592 | No |
| 17 | HLA-DMA | na | 17342 | -0.006 | -0.1978 | No |
| 18 | HLA-DOB | na | 17625 | -0.008 | -0.2043 | No |
| 19 | HLA-A | na | 17972 | -0.010 | -0.2122 | No |
| 20 | HLA-B | na | 19514 | -0.021 | -0.2522 | No |
| 21 | CD28 | na | 19606 | -0.021 | -0.2503 | No |
| 22 | HLA-DRB3 | na | 19647 | -0.021 | -0.2469 | No |
| 23 | TNF | na | 22702 | -0.047 | -0.3249 | No |
| 24 | HLA-DQA2 | na | 24037 | -0.063 | -0.3499 | No |
| 25 | FAS | na | 24099 | -0.064 | -0.3381 | No |
| 26 | CD40 | na | 26388 | -0.096 | -0.3835 | No |
| 27 | IL12B | na | 26869 | -0.106 | -0.3748 | No |
| 28 | HLA-DMB | na | 27501 | -0.119 | -0.3676 | No |
| 29 | IL2 | na | 30766 | -0.213 | -0.4162 | Yes |
| 30 | HLA-DQA1 | na | 30949 | -0.219 | -0.3748 | Yes |
| 31 | CD80 | na | 31382 | -0.235 | -0.3371 | Yes |
| 32 | CD40LG | na | 31605 | -0.244 | -0.2915 | Yes |
| 33 | IL5 | na | 32563 | -0.292 | -0.2568 | Yes |
| 34 | IL10 | na | 32834 | -0.310 | -0.1984 | Yes |
| 35 | IL4 | na | 33709 | -0.380 | -0.1424 | Yes |
| 36 | IFNG | na | 33859 | -0.398 | -0.0618 | Yes |
| 37 | PRF1 | na | 33908 | -0.407 | 0.0236 | Yes |
Table: GSEA details [plain text format]

  

Fig 2: KEGG\_ALLOGRAFT\_REJECTION      
 Blue-Pink O' Gram in the Space of the Analyzed GeneSet

  

Fig 3: KEGG\_ALLOGRAFT\_REJECTION: Random ES distribution      
 Gene set null distribution of ES for **KEGG\_ALLOGRAFT\_REJECTION**
